# Supplementary material for: Effect of dietary inclusion of artichoke (Cynara scolymus) leaf powder and astaxanthin on productive performance, hematological and biochemical profiles, hepatic and antioxidant enzyme activities, and gut microbiota in broiler chickens
Source: PLoS One. 2025 Oct 24;20(10):e0328784. doi: 10.1371/journal.pone.0328784 (PMC12551905; doi:10.1371/journal.pone.0328784)
Supplement: Attachment — Submitted filename: Response to Reviewers.docx [file pone.0328784.s004.docx]

**Reviewer #1:** While the manuscript presents a well-designed experiment exploring the use of herbal additives to prevent FLHS in laying hens, the overall quality of English language and expression requires significant improvement. Numerous grammatical errors, awkward phrasings, and formatting inconsistencies—especially in the introduction and methods sections—may hinder reader comprehension. For instance, several sentences are either incomplete or improperly constructed, such as line 76–77, and citation formatting is occasionally unclear or broken. I recommend that the authors undergo thorough professional English language editing to enhance clarity, readability, and overall presentation before further consideration.

We have thoroughly reviewed the entire manuscript and carefully revised the text to address all grammatical errors, awkward phrasings, and formatting inconsistencies, particularly in the Introduction and Methods sections as noted. The sentence on lines 76–77 has been restructured for clarity. The citation formatting throughout the manuscript has been corrected. We have also sought assistance from a professional English language editing service to ensure the manuscript meets high standards of readability and presentation.

Line 37, the author could change “background” to “introduction”, because “background” may be confused as the same one in Abstract.

The heading “Background” was changed to “Introduction”.

Lines 38–42: The point that the liver synthesizes lipids and is heavily burdened in hens is repeated. The author could try to combine them with a single sentence.

We have revised the text to eliminate redundancy and merged the information into a concise sentence (page 2, lines 40-44).

The methods mentioned in the manuscript should be cited formally. Such as Line 51-54.

We have carefully reviewed the entire Methods section to ensure all procedures, including those mentioned in lines 51–54, are properly cited with relevant and appropriate references.

Lines 55–64: The paragraph listing all plants and their bio-actives is dense and hard to read. The author could Use a table or bullet points for readability or briefly summarize: "These herbs are rich in flavonoids, alkaloids, sulfur compounds, and dietary fibers, with demonstrated antioxidant, anti-inflammatory, and lipid-lowering properties."

Thank you for your helpful suggestion. We have replaced the dense paragraph with a table summarizing the medicinal plants used and their key bioactive components (Table 1).

Lines 37-69, the author could use clearer topic sentences to guide the reader through each section logically, including liver function, disease description, economic impact, and herbal solutions.

We have revised lines 37–69 to include clearer and more distinct topic sentences at the beginning of each major section.

Lines 88–90, the author use informal phrasing “running into the experimental trial", which can be corrected to "A two-week adaptation period was implemented to acclimate hens to the diets prior to the 12-week experimental trial."

We have revised the sentence to eliminate informal phrasing.

For animal study design, the randomization or blinding details were missing. Please mark those in the experiment design part.

Thank you for highlighting this important point. In response, we have updated the experimental design section to include details regarding randomization and blinding (page 5, lines 89-90).

No mention of sample size calculation or statistical power. The author could revise as "Sample size was determined based on expected effect sizes from previous studies to achieve 80% power at α = 0.05."

We have revised the Methods section to include a statement on sample size calculation. The text now reads: *“Sample size was determined based on expected effect sizes from previous studies to achieve 80% power at α = 0.05”* (page 5, lines 88-89).

**Reviewer #2:** Dear Authors, Strictly follow my comments/corrections in the attached file.
1. In the introduction section, need for the study should be highlighted at the end followed by the hypothesis of the study

Thank you for your insightful comment. We have revised the final part of the Introduction to clearly emphasize the need for the study and to better establish the context and significance of our research. Additionally, we have now explicitly stated the hypothesis of the study to guide the reader and clarify our research objectives.

2. In methodology, clearly elaborate the sample size determination

We have revised this section to provide a clear explanation of how the sample size was determined (Page 5, lines 88-89).

3. Add appropriate citations

We have thoroughly reviewed the entire Methods section and ensured that all procedures are now properly cited with relevant and appropriate references.

4. In the results, improve readability and add actual p-value of each result

Thank you for your valuable comment. We have thoroughly revised the Results section to enhance clarity and improve readability. Additionally, we have included the actual p-values for all statistical comparisons rather than simply stating significance levels (e.g., *p* < 0.05).
5. In the discussion, add logical reasoning of each result before discussing with the previous studies.

Thank you. We have revised the Discussion section to provide a clear and logical interpretation of each key result before comparing our findings with those of previous studies. These revisions aim to enhance the scientific depth and coherence of the discussion.

6. In the conclusion, briefly describe and add limitation of the study.

We have revised the Conclusion section to include a brief summary of the main findings, emphasizing the significance of our results. In addition, we have acknowledged the limitations of the study, such as the relatively short duration, the use of a single dose per herbal mixture, the lack of individual herb evaluation, and the restriction to one hen strain and age group. This addition provides a balanced perspective and suggests directions for future research.

Ad libitum feed was provided? at this age? why?

The CD was intentionally formulated to predispose hens to fatty liver hemorrhagic syndrome (FLHS). However, due to its imbalanced nutrient profile, feed intake could be influenced as hens attempt to compensate for nutrient deficiencies. By allowing *ad libitum* access, we enabled the hens to express natural compensatory feeding behavior, which is crucial for observing true diet-induced metabolic and hepatic changes.
